# Conferencing well

**DOI:** 10.1007/s40037-022-00704-0

**Published:** 2022-03-03

**Authors:** Glenn Regehr, Lara Varpio

**Affiliations:** 1 Centre for Health Education Scholarship, Faculty of Medicine, University of British Columbia, Vancouver, Canada; 2 Department of Medicine, Uniformed Services University of the Health Sciences, Bethesda, MD USA

In the Insider’s Perspective section, an insider in health professions education offers his/her thoughts, contemplations and advice on readers’ dilemmas or questions. You can send your questions or dilemmas to lieda.meester@bsl.nl. And who knows, your question may be the topic of the next instalment of the Insider’s Perspective.

*Dear Insider, I am just starting my career as a clinician educator. I am told that participating in our field’s academic conferences is important. Any tips for how I can get the most out of my conference experience?*

Conferences are more than just a place where scholars present their research and listen to others present theirs. Academic conferences are extraordinary events. It is not ordinary to be within an arm’s length of so many members of your academic community. It is not ordinary to attend mixers where attendees aim to greet long-standing collaborators and to meet new ones. It is not ordinary to engage in direct conversations with those whose work you have been reading for years or to be approached by those who have been reading your work. As your question suggests, the trick is to figure out how to take advantage of these extraordinary circumstances.

Before we dive in, we offer a few premises that guide our thinking about what it means to “conference well”. First, our approach to conferencing has largely been shaped by our own experiences. As it has with so many aspects of daily life, the Covid-19 pandemic has radically altered the community’s model of conferencing. Whether this is a permanent change or merely a brief disruption to the traditional approach of in-person conferencing remains to be seen. But, in truth, we are still learning how to effectively engage in web-based conferences. Thus, the approaches for “conferencing well” that we share here are derived from in-person conference experiences, recognizing that as conference formats change, practices will also have to evolve.

Second, we believe that the conference’s formal program is an important component of the conference experience, and reviewing the program to identify the sessions and people that you want to hear is time well spent. However, these programs are often large, complicated, and difficult to navigate. It isn’t easy to tell from the program descriptions which sessions you will find particularly enlightening nor which will be “must see” sessions. We suggest looking for a few promising sessions and checking in with others to see what they think is particularly promising. However, if all you get out of the conference are a few great formal sessions, then you will not have taken full advantage of what the conferencing situation affords.

So, then, what do (in-person) conferences afford? Science is a social enterprise, and a conference is a social activity. Conferences enable you to do quickly the things that academia generally does very slowly. For example, the journey from cool idea to journal publication can take a long time. So, if you are reading the journals as your way of staying current, then you are at least two to three years out of date regarding what people are really thinking about. Conferences are where people are talking about what they are presently considering. But even listening to the formal talks at the conference likely means you are at least a year out of date. The ideas that people are evolving right now can only be discovered if you engage with them individually through informal conversations in hallways or cafés. So, these casual conference conversations are a key mechanism to stay current with the cutting-edge ideas in the field.

Such informal conference conversations also allow you to develop and disseminate your own cutting-edge ideas more rapidly. The academic review process is slow and can be spotty. It can be frustrating to wait 3–6 months on the review of your submitted paper only to receive reviews expressing vague concerns that don’t seem to make sense or that require further clarification to make actionable. Thus, having informal conversations with colleagues at conferences is a great way to quickly and effectively share your ideas and get immediate insight into others’ reactions to your latest thinking and work. How will others interpret your explanations? How will they react to your framings? Who else might be interested in or doing similar work? The informal conversations at a conference are where you can get immediate answers to these kinds of questions from your peers.

Further, developing your reputation as a thoughtful scholar is difficult to accomplish through the written word alone. Simply doing good science and writing good papers may not be sufficient to get noticed and influence the community. If you want people to pick up your work, it is helpful to give them a reason to look for it and to help them understand how it is relevant to their ideas. This is best done face-to-face in active discourse, and conferences are a great place to have these conversations with people you want to influence and engage with academically. And, as a corollary, through thoughtful engagement with people about *their* early ideas, you can develop a reputation for supporting and enhancing others’ work (which, by the way, may increase the likelihood that you will hear about people’s current thinking, as well as open the door for research collaborations). So going out of your way to meet new people is not only about extending your friendship group, but it is also about extending your network and sphere of influence.

Finally, conferences should be fun and refreshing places, but they are also where you are constructing your professional image. In academia, the coin of the realm is your name, your reputation. It is fundamentally important to guard your image and to nurture it intentionally. So absolutely take some personal time to see the sights and enjoy the location (as one colleague says, “one should return from a conference better rested and better dressed”). And, of course, go out for social dinners and drinks with friends and colleagues at the conference. But it is also important to remember that the conference is a professional event where you represent yourself, your work, and your institution.

So how do these ideas play out in actual conference preparations and activities? What can you do to maximize the effectiveness of your conference activities?

A good start is having a plan to meet specific people. We all have people we want to connect with in person—some are collaborators we are actively working with or friends we haven’t seen for a while. Others are people we’d like to introduce ourselves to. Having a list of the people you want to meet at the conference is a great first step to making these connections. You might wish to contact some of these individuals ahead of the event and plan a specific meeting time. Ideally, this works best if you contact them relatively soon after the conference schedule has come out. This is the window during which they know their program obligations (i.e., sessions they must attend), but their non-obligatory conference time will not have filled up yet. For friends and established colleagues, you might arrange meals together. For acquaintances, you might arrange times between meals to keep the meetings shorter. For those you are hoping to meet for the first time, you might leave the request more open, simply asking if they might have a bit of free time in their schedule for a brief meeting. Notably, if you are hoping to meet people for the first time, it is advisable to have a specific agenda for these meetings. A reasonable agenda can be simply to make the other person aware of your work and to ask for their reaction. An agenda that tends to fall flat is: “I just want you to know who I am.”

For other people, you may wish to make the connection more informally, finding a way to bump into them at the conference. Although you might approach them at the end of a session or between sessions in the hallways, such ad hoc meetings can happen most easily at the conference activities specifically planned for casual social networking. The receptions, buffet breakfasts, unstructured poster sessions, and other meet-and-greet events are often when those you are hoping to introduce yourself to are specifically expecting these spontaneous connections. If it feels intimidating to walk up to people in this way, then one option is to arrange for a mutual acquaintance to make introductions on your behalf. And this raises another noteworthy point: When you’re new to the field, having someone senior to sponsor you at the conference and help you make connections with other community members is a real advantage. To that end, arranging (in advance of the conference) to spend time beside a senior colleague or mentor at a particular mixer event and asking them to introduce you to key individuals can help you connect with people on your “*to meet*” plan and with others you hadn’t recognized as important connections. As a side note to more senior members of the field: one of the most valuable things we feel we do at a conference is sponsor and promote one or two early-career colleagues. So, as we plan for our conference, we ask ourselves who will be shadowing us at the meet-and-greet events.

Harnessing these more informal meetings to your advantage requires preparation. For example, you will want to have answers prepared for the inevitable questions: “Can you tell me about yourself? What do you do?” Often called the *elevator pitch*, having a very short (30 second) and a little longer (2 minute) answer for these questions practiced and ready for delivery can be incredibly helpful. In the busy social context of conferences, you will have a small window of time to situate yourself in the field and give the listener a sense of what is unique and interesting about your work. This is not the time to stumble or start with a vague reply like, “it’s complicated.” Practicing these answers before you get to the conference will ensure you don’t stumble when you are introduced to someone you’ve wanted to meet. Remember, conferences are where things happen quickly. You will be crafting your social capital in those short informal conversations. In a matter of minutes, the listener will form their impressions of you and your work. Make those minutes count by practicing your answers with trusted colleagues before you leave for the conference and ask for feedback: Can you summarize what you understood from what I said? Was it interesting? Did I manage to convey why this work is important?

In addition to this focus on introducing yourself and sharing your ideas, however, don’t forget to be useful to others as well. Be a helpful audience for the ideas that others are trying to test. Offer theories, perspectives, or ideas that might be relevant to their work. Share other literature they might not have come across yet. Connect them with (e.g., offer to introduce them to) others you know exploring related ideas. Whether these interactions happen in the context of an informal meeting with someone, through a brief conversation in the context of their poster, or as a quick one-minute exchange following their formal presentation on the podium, offering interesting leads and giving away related ideas can create a reputation for being generous and generative, which is the sort of reputation that is never too early to start developing.

Finally, remember to follow up after (or during) the conference with some form of electronic connection to keep the relationship alive. If you mentioned an article they might wish to read or an author whose work they might wish to explore, send them an electronic version of the paper (or a few specific references of the relevant author). If you promised to make a connection with someone, send the e‑introduction as soon as you can. If you were sharing some of your work with them, send them an electronic version of your article, or even a draft if you (and your supervisor or mentor) feel it is close enough for external eyes to see. If they offered you something, email them to thank them for the offer as a way of reminding them. Or if it was just a nice conversation, just follow up with a quick note to say how nice it was to meet them. A quick trick in this regard, by the way, is to make a note to yourself immediately after meeting with a person regarding what you discussed and what you offered. We all make conference promises, many of which get forgotten almost immediately after they were made. So having a strategy to ensure you remember and follow up on these valuable conversations is essential if you wish to maintain that connection. We used to make notes of these conversations on the back of the person’s business card; nowadays, we make notes and reminders in our phones. *How *you follow up is up to you; just make sure you *do *it.

There are many potential positive outcomes of “conferencing well”: having refined and revised your thinking about a project; having new ideas that you hadn’t previously considered and new authors to explore; having given away a few ideas that others are more likely to follow up on than you; having reconnected with old colleagues and made some new connections; as well as having some new pictures in your phone from places visited; having more energy because you are better rested than when you arrived; and having luggage that is a little heavier with souvenirs and other purchases. Conferencing well is an art that can take some time to hone, but it is well worth the effort to ensure that you can take advantage of these extraordinary events.

